# Inhibition of microRNA-146a attenuated heart failure in myocardial infarction rats

**DOI:** 10.1042/BSR20191732

**Published:** 2019-12-23

**Authors:** Junjie He, Ying Lu, Xiaozheng Song, Xiaoxuan Gong, Yong Li

**Affiliations:** 1 Lianshui County People’s Hospital, Huaian, China; 2 Department of Laboratory Medicine, The First Affiliated Hospital of Nanjing Medical University, Nanjing, China; 3 Department of Cardiology, Shengli Oilfield Central Hospital, Dongying, China; 4 Department of Cardiology, the First Affiliated Hospital of Nanjing Medical University, Nanjing, China

**Keywords:** myocardial infarction, heart failure, microRNA-146a, cardiac dysfunction, cardiac remodeling

## Abstract

The aim of the present study was to determine the roles of microRNA (miR)-146a on myocardial infarction (MI)-induced heart failure and cardiac remodeling. Experiments were carried out in Sprague-Dawley rats treated with ligation of left coronary artery to induce heart failure, and in primary neonatal rat cardiac fibroblasts (CFs) and cardiomyocytes treated with angiotensin (Ang) II. Four weeks after MI, rats were injected with miR-146a antagomiR or agomiR via tail vein. After 2 weeks of injection, the rats were killed. In MI rats, left ventricle (LV) ejection fraction and fractional shortening were reduced, and LV volumes in diastole and systole were increased, which were reversed by miR-146a antagomiR, and further exacerbated after miR-146a agomiR treatment. Administration of miR-146a antagomiR improved the decreases of LV ±d*p*/d*t*_max_ and LV systolic pressure (LVSP), and the increase in LV end-diastolic pressure (LVEDP) of MI rats, but miR-146a agomiR deteriorated the LV ±d*p*/d*t*_max_, LVSP and LVEDP. The increases in the levels of atrial natriuretic peptide (ANP), brain natriuretic peptide (BNP), collagen I and collagen III in the heart, and ST2 and norepinephrine in the serum of MI rats were inhibited by miR-146a antagomiR, but aggravated after miR-146a agomiR treatment. The increases of collagen I and collagen III levels induced by Ang II in CFs, and the increases of ANP and BNP levels induced by Ang II in cardiomyocytes were inhibited by miR-146a antagomiR, but aggravated by miR-146a agomiR. These results demonstrated that inhibition of miR-146a improved cardiac dysfunction and cardiac remodeling in heart failure rats.

## Introduction

The prevalence of heart failure (HF) continues to rise over time with the aging population [1]. HF, a complex syndrome defined by inadequate cardiac output to meet the metabolic demands of body organs, resulted from a wide range of congenital and acquired cardiovascular or metabolic diseases leading to structural and functional cardiac impairment [2,3]. Epidemiological studies have indicated numerous causative and risks factors of HF, including coronary artery disease, diabetes mellitus, valvular heart disease, hypertension, atrial fibrillation and obesity [4,5].

HF is preceded by adverse LV remodeling (LV hypertrophy, LV dilatation, LV sphericity), as demonstrated in rats [6], rabbits [7], pigs [8,9] and humans [10]. Atrial natriuretic peptide (ANP), brain natriuretic peptide (BNP), collagen I and collagen III levels were increased in HF [7].

MicroRNAs (miRs) are a group of small non-coding RNAs, which serve as negative regulators for target gene expressions mainly through translation inhibition or mRNA degradation [11–14]. The understanding and discovery of miRNAs has raised the possibility of using circulating miRNA as biomarkers in cardiovascular disease (CVD) [15,16]. Increasing evidence has demonstrated that several miRNAs, including miR-146a, miR-190a, miR-1233, miR-193b-3p, miR-211-5p are dysregulated in human or experimental model with heart failure [4,17]. However, the relevant mechanisms are far from understood.

Study revealed that miR-146a could be used as potential biomarkers for evaluating the efficacy of anti-heart failure drugs [18]. *In vivo* transfection of lentivirus-expressing miR-146a (LmiR-146a) attenuated sepsis-induced cardiac dysfunction, and the values for percent ejection fraction (EF) and percent fractional shortening (FS) in LmiR-146a-transfected cecal ligation and puncture (CLP) mice were significant greater than in untransfected CLP control [19]. Overexpression of miR-146a reduced cardiac contractility *in vitro* and *in vivo* [20]. However, the effects of miR-146a on heart failure and cardiac remodeling in myocardial infarction (MI) are unknown. The present study was to determine whether inhibition miR-146a attenuated cardiac dysfunction and cardiac remodeling, and the signaling pathway in MI-induced heart failure rats.

## Materials and methods

### Animals

Experiments were carried out with 160–180 g male Sprague-Dawley (SD) rats (Vital River Biological Co., Ltd, Beijing, China). The rats were kept in a temperature-controlled room on a 12 h light–dark cycle with free access to standard chow and tap water. All procedures were approved by the Experimental Animal Care and Use Committee of Nanjing Medical University (Nanjing, China), and were conducted in accordance with the Guide for the Care and Use of Laboratory Animals (NIH publication No. 85-23, revised 1996). The related experiments were conducted at Experimental Animal Center of Nanjing Medical University. The ethical approval number is 14051386 and was approved in 2017.

### Myocardial infarction model

Myocardial infarction is a common cause in driving the occurrence of the left ventricular dysfunction and heart failure. The myocardial infarction rats in present study were induced by coronary artery ligation with sterile techniques as previously reported [21]. Briefly, the rats were anesthetized with sodium pentobarbital (50 mg kg^−1^, i.p.). The rats were randomly subjected to the ligation of the left anterior descending coronary artery and sham-operated (Sham) groups. The heart was exposed through a left intercostal thoracotomy, and left coronary artery was looped by a single nylon suture. Finally, the heart was quickly repositioned into the chest. The Sham rats were treated the same as the coronary ligation rats except that their coronary arteries were not ligated.

### Echocardiography

Transthoracic echocardiography was performed under isoflurane anesthesia using an ultrasound system (Vevo 2100, VisualSonics, Toronto, Canada) with a 21-MHz probe. The left ventricular end-diastolic diameter (LVEDD) and end-systolic diameter (LVESD), and LV volumes in diastole (LVVd) and systole (LVVs) were measured. The LV ejection fraction (EF) and fractional shortening (FS) were calculated. Measurements over three consecutive cardiac cycles were averaged.

### Hemodynamic monitoring

Rats were anesthetized with isoflurane and a 1.4F conductance micromanometer catheter (Millar Instruments, TX, U.S.A.) was inserted into the LV chamber via the right carotid artery across the aortic valve. The maximum of the first differentiation of left ventricular pressure (LV +d*P*/d*t*) and decline (LV −d*P*/d*t*), left ventricle systolic pressure and LV end-diastolic pressure (LVEDP) were obtained on a PowerLab data acquisition system (AD Instruments, Sydney, Australia).

### Quantitative real time-PCR (qRT-PCR)

The total RNA in samples was extracted with TRIzol (Ambion, TX, U.S.A.). cDNA was extracted from the RNA with reverse transcription using random primers in a total volume of 10 μl according to the instructions of the PrimeScript™ RT Master Mix (Takara, China). All cDNA were stored at − 70°C before used. ANP, BNP, collagen I and collagen III mRNA were determined with SYBR Green I fluorescence. All samples were amplified in triplicates for 45 cycles in a 96-well plate. The relative gene expression was determined by calculating the values of Δcycle threshold (Δ*C*_t_) as a relative quantity to the endogenous control. The primers are shown in Table 1.

**Table 1:** List of utilized primers for qRT-PCR

Gene	Species	Forward primer	Reverse primer
ANP	Rat	GAGCAAATCCCGTATACAGTGC	ATCTTCTACCGGCATCTCCTCC
BNP	Rat	GCTGCTGGAGCTGATAAGAGAA	GTTCTTTTGTAGGGCCTTGGTC
Collagen I	Rat	TCAAGATGGTGGCCGTTAC	CTGCGGATGTTCTCAATCTG
Collagen III	Rat	CGAGATTAAAGCAAGAGGAA	GAGGCTTCTTTACATACCAC
GAPDH	Rat	GGCACAGTCAAGGCTGAGAATG	ATGGTGGTGAAGACGCCAGTA

Abbreviations: ANP, atrial natriuretic peptide; BNP, brain natriuretic peptide.

### MiRNA-146a antagomiR and agomiR injections in rats

To determine the functional role of miR-146a in MI-induced heart failure, 4 weeks after MI, rats were injected with miR-146a antagomiR (a 2′OME+5′chol modified miR-146a inhibitor, 80 mg/kg/day) or agomiR (a 2′OME + 5′chol modified miR-146a agonist, 30 mg/kg/day) via tail vein for three consecutive days. miR-146a antagomiR and agomiR were synthesized by Ribobio Co. (Guangzhou, China). The sequences of miR-146a agomiR are as follows: 5′-UACGCCCUUUUAACAUUGCAUCG-3′. The antagomiR is a singles-tranded RNA analog complementary to the mature miR-146a, which is chemically modified and cholesterol conjugated from a hydoxyprolinol linked cholesterol solid support and 2′-Ome phosphoramidites. After 2 weeks of injection, the rats were killed.

### ST2 and norepinephrine levels determination

Serum ST2 and norepinephrine (NE) were measured with ELISA kits (Cloud-Clone Corp., Wuhan, China) according to the manufacturer’s descriptions. The 96-well plates were incubated with antibodies specific for rat ST2 or NE, respectively. Standard diluent buffer and samples were added, incubated and washed. Horseradish peroxidase-conjugated solution was added and then washed out. The reactions were stopped with stop solution and the final solution was read at 450 nm using a microplate reader (BioTek, VT, U.S.A.).

### Sirius Red staining

Cardiac sections (5 µm) were examined Sirius Red staining (Nanjing Biochannel Biotechnology Co., Ltd, Nanjing, China), according to the manufacture instruments to measure the fibrosis of heart. Three to five random fields were selected from each of three sections from each animal for observation under a light microscope (Carl Zeiss GmbH, Oberkochen, Germany). Images were analyzed using Image-Pro Plus software (Media Cybernetics, Inc., MD, U.S.A.).

### Western blotting

Heart samples were sonicated in RIPA lysis buffer and homogenized. The debris was removed and the supernatant was obtained by centrifugation at 12,000 *g* for 10 min at 4°C. Approximately 30–50 μg protein was separated by electrophoresis, transferred to PVDF membrane, and probed with primary antibodies against Akt, p-Akt, ERK and p-ERK (Cell Signaling, MA, U.S.A.); and GAPDH (Abcam, MA, U.S.A.) as an internal control. Images were analyzed using Image-Pro Plus software.

### Culture of cardiomyocytes isolated from neonatal rat

Primary cardiomyocytes were isolated from 1- to 3-day-old newborn Sprague-Dawley rats (Vital River Biological Co.). Hearts were excised and digested in PBS (Nanjing Biochannel Biotechnology Co., Ltd) containing collagenase type II (Worthington Biochemical Corp., NJ, U.S.A.) and pancreatin (Sigma, MO, U.S.A.). The atria and great vessels were discarded. The ventricles were cut into small pieces and further digested with collagenase type II and pancreatin. Cells from digestion were collected and cultured in Complete Dulbecco’s modified Eagle’s medium (DMEM, Nanjing Biochannel Biotechnology Co., Ltd) including 10% fetal bovine serum (FBS, Nanjing Biochannel Biotechnology Co., Ltd) for 2–4 h to reduce fibroblasts and enrich for cardiomyocytes. The cardiomyocytes were cultured at 37°C with 5% CO_2_. Cardiomyocytes were incubated with 10^−6^ M Ang II (Sigma, St Louis, MO, U.S.A.) for 24 h, and treated with miR-46a antagomiR or agomiR according to the manufacturers’ instructions.

### Isolation and culture of cardiac fibroblasts (CFs)

Rat cardiac fibroblasts (CFs) were isolated from 1- to 3-day-old SD rats. Briefly, CFs were separated from cardiomyocytes by gravity separation and grown to confluence on 10-cm cell culture dishes with growth media (DMEM including 10% FBS, 1% penicillin and 1% streptomycin) at 37°C in humid air with 5% CO_2_ and 95% O_2_. CFs from the second passage were used for the subsequent experiments. CFs were incubated with 10^−6^ M Ang II (Sigma, St Louis, MO, U.S.A.) for 24 h to induce the fibrotic phenotype, and treated with miR-46a antagomiR or agomiR according to the manufacturers’ instructions.

### Statistical analyses

Data are presented as mean ± standard error of the mean (SEM). Using GraphPad Prism 4.0 (GraphPad software Inc., CA, U.S.A.), statistical significance among multiple groups was evaluated by one-way analysis of variance (ANOVA) with the Bonferroni post-hoc test. A two-tailed *P*-value <0.05 was considered statistically significant.

## Results

### Effects of miR-146a on cardiac function in MI rats

**Figure 1:**
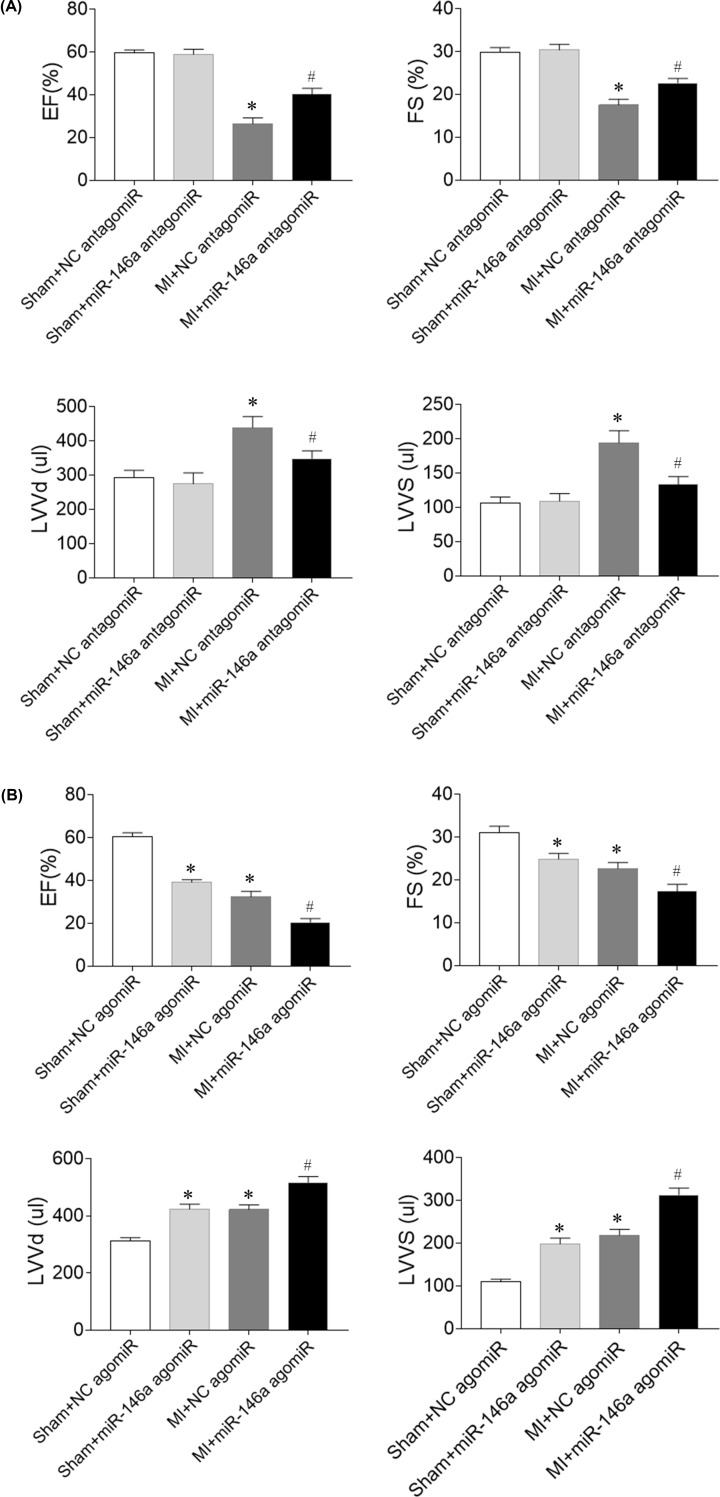
Effects of microRNA (miR)-146a on cardiac dysfunction of myocardial infarction (MI)-induced heart failure (HF) rats (**A**) miR-146a antagomiR improved cardiac dysfunction of HF rats. (**B**) miR-146a agomiR exacerbated cardiac dysfunction of HF rats. The results are expressed as mean ± SEM; *N* = 6. *P < 0.05 versus the Sham group; ^#^*P* < 0.05 versus the MI group.

Echocardiography showed that MI induced reduction of EF (%) and FS (%), and increases of LVVd and LVVs. For three consecutive days, miR-146a antagomiR injection via tail vein increased the reduction of EF (%) and FS (%), and reduced the increases of LVVd and LVVs in heart failure rats (Figure 1A). Treatment with miR-146a agomiR reduced EF (%) and FS (%), and increased LVVd and LVVs in Sham rats. miR-146a agomiR aggravated the decreases of EF (%) and FS (%) in MI rats. Furthermore, the increases of LVVd and LVVs in MI rats were further enhanced by miR-146a agomiR injection (Figure 1B).

### Effects of miR-146a on cardiac hemodynamics in MI rats

**Figure 2:**
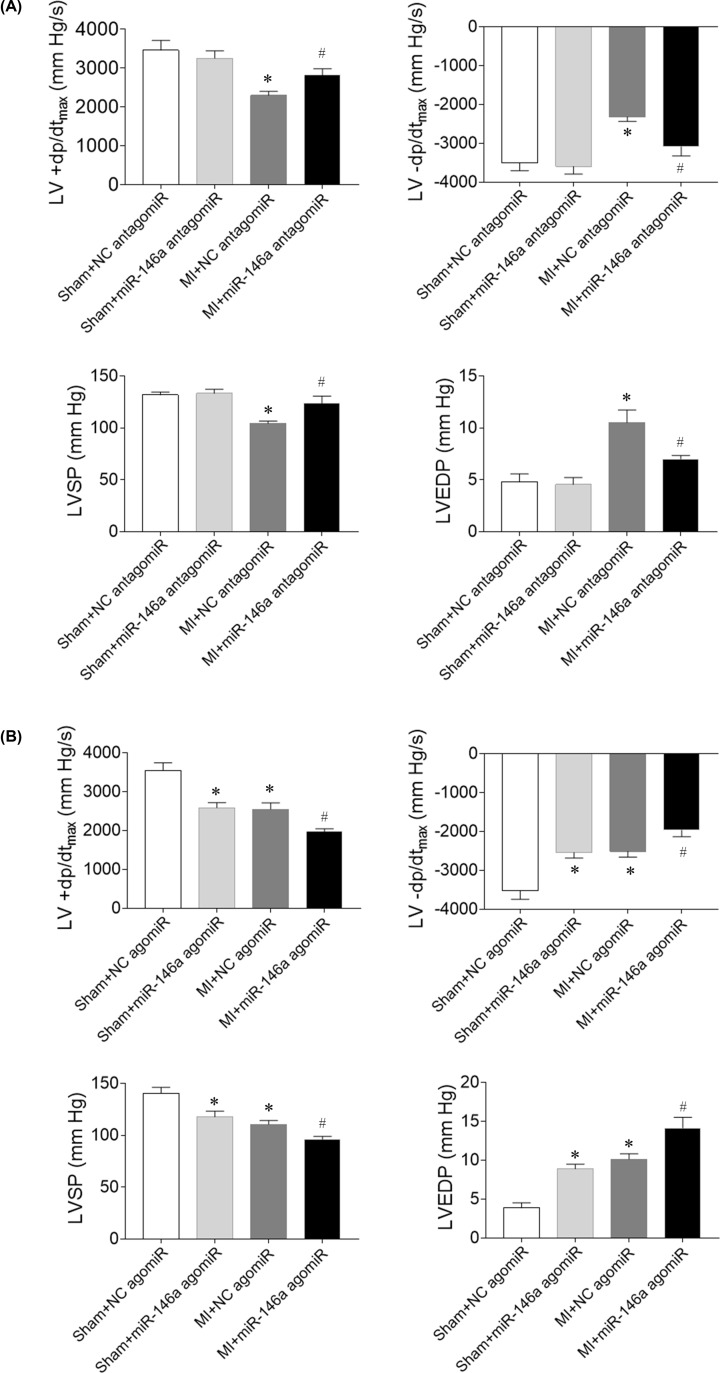
Effects of microRNA (miR)-146a on impaired cardiac hemodynamics of myocardial infarction (MI)-induced heart failure (HF) rats (**A**) miR-146a antagomiR improved impaired cardiac hemodynamics of HF rats. (**B**) miR-146a agomiR exacerbated impaired cardiac hemodynamics of HF rats. The results are expressed as mean ± SEM; *N* = 6. *P < 0.05 versus the Sham group; ^#^*P* < 0.05 versus the MI group.

LV +d*p*/d*t*_max_, LV −d*p*/d*t*_max_ and LVSP were decreased in MI rats, and miR-146a antagomiR injection increased the decreases of LV +d*p*/d*t*_max_, LV −d*p*/d*t*_max_ and LVSP induced by MI. LVEDP was increased in the heart failure rats induced by MI, which was reversed by miR-146a antagomiR treatment (Figure 2A). miR-146a agomiR reduced LV +d*p*/d*t*_max_, LV −d*p*/d*t*_max_ and LVSP in Sham rats. LVEDP was increased after miR-146a agomiR injected into Sham rat. Injection of miR-146a agomiR into the vein aggravated the decreases of LV +d*p*/d*t*_max_, LV −d*p*/d*t*_max_ and LVSP. The increase of LVEDP was reinforced by miR-146a agomiR injection (Figure 2B).

### Effects of miR-146a on neurohormonal activation in MI rats

**Figure 3:**
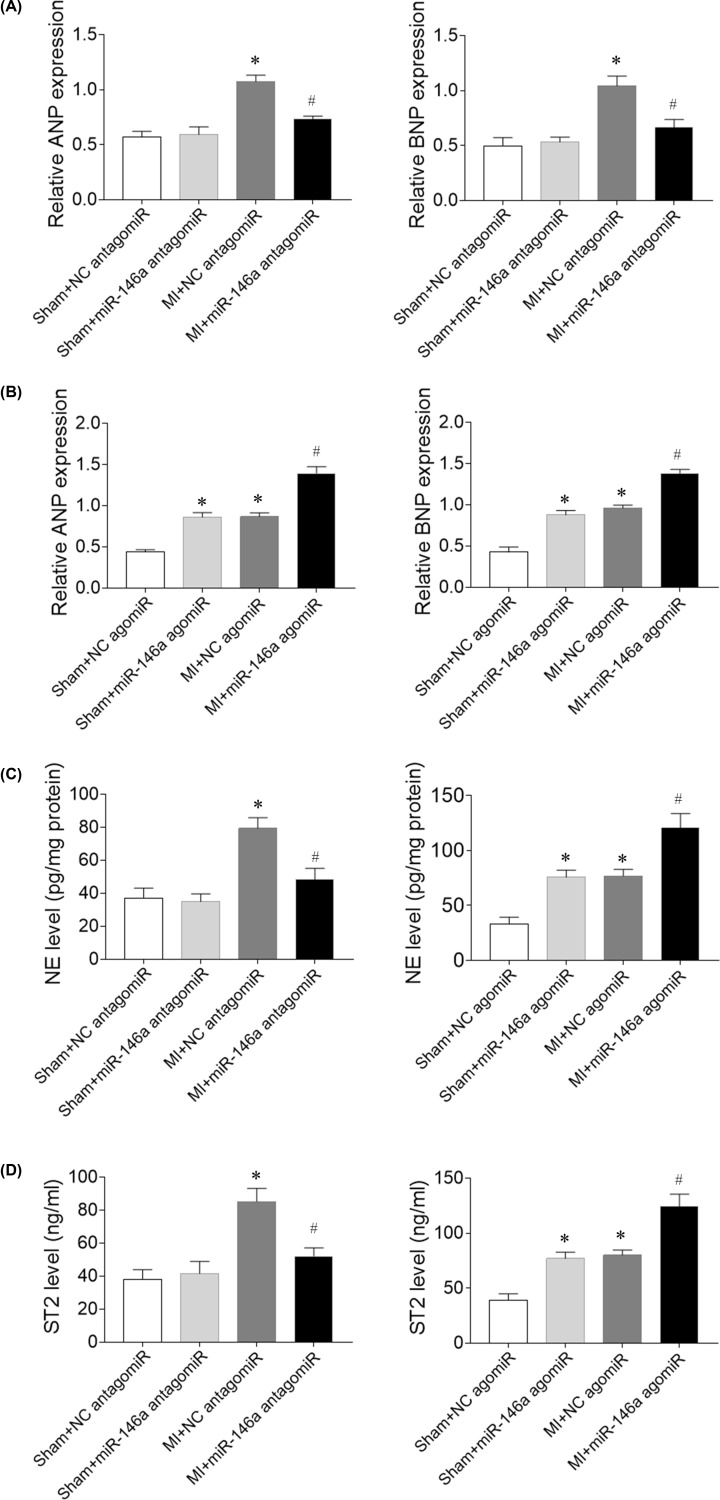
Effects of microRNA (miR)-146a on neurohormonal activation in myocardial infarction (MI)-induced heart failure (HF) rats (**A**) The increases of atrial natriuretic peptide (ANP) and brain natriuretic peptide (BNP) mRNA levels were reduced after miR-146a antagomiR treatment in MI rats. (**B**) The increases of ANP and BNP mRNA levels were further enhanced after miR-146a agomiR treatment in MI rats. (**C**) The levels of ST2 were attenuated by miR-146a antagomiR and were further worsen by miR-146a agomiR treatment in MI rats. (**D**) The levels of norepinephrine (NE) were attenuated by miR-146a antagomiR and were further worsen by miR-146a agomiR treatment in MI rats. The results are expressed as mean ± SEM; *N* = 6. *P < 0.05 versus the Sham group; ^#^*P* < 0.05 versus the MI group.

The levels of ANP and BNP mRNA in the heart were higher in MI rats than that in sham rats. The increase of ANP and BNP mRNA levels were reduced after miR-146a antagomiR treatment (Figure 3A). Treatment with miR-146a agomiR increased ANP and BNP mRNA levels in the heart of Sham rats. The increases of ANP and BNP mRNA levels were further enhanced after miR-146a agomiR treatment in MI rats (Figure 3B). The levels of ST2 and NE levels were higher in the serum of MI rats than that in sham rats, which were attenuated by miR-146a antagomiR and were further worsen by miR-146a agomiR treatment (Figure 3C,D).

### Effects of miR-146a on cardiac fibrosis in MI rats

**Figure 4:**
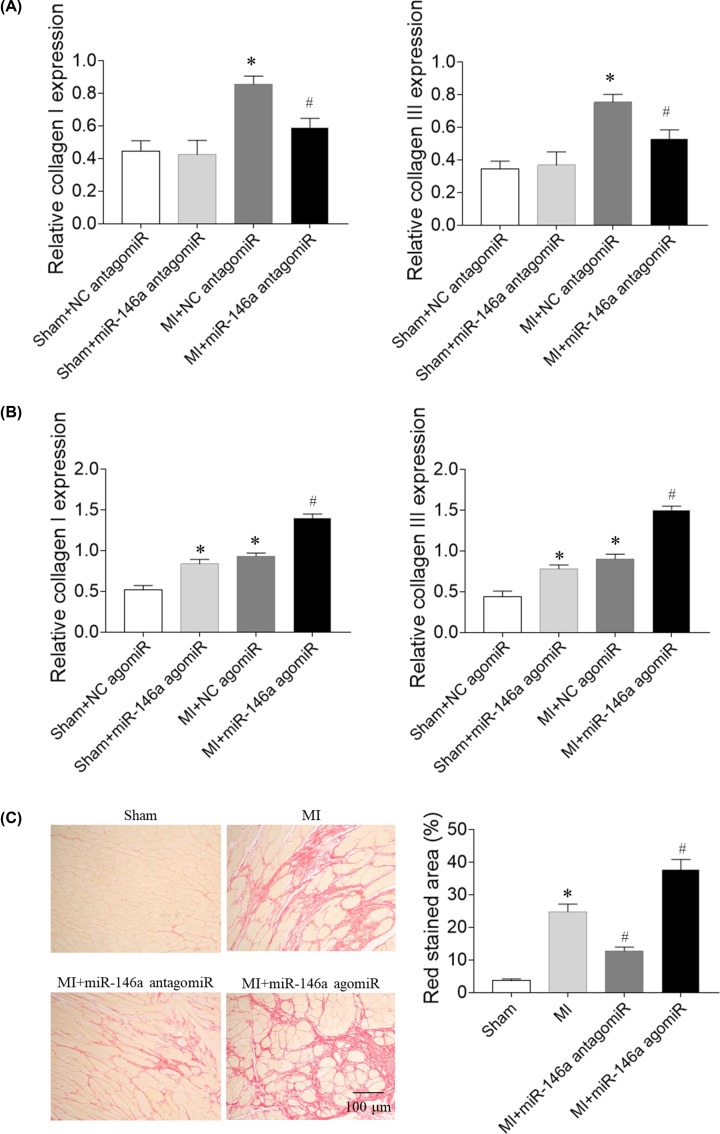
Effects of microRNA (miR)-146a on cardiac fibrosis of myocardial infarction (MI)-induced heart failure (HF) rats (**A**) miR-146a antagomiR reduced collagen I and collagen III mRNA levels in HF rats. (**B**) miR-146a agomiR increased collagen I and collagen III mRNA levels in HF rats. (**C**) miR-146a antagomiR attenuated cardiac fibrosis of HF rats, and miR-146a agomiR exacerbated cardiac fibrosis of HF rats. The results are expressed as mean ± SEM; *N* = 6. *P < 0.05 versus the Sham group; ^#^*P* < 0.05 versus the MI group.

The levels of collagen I and collagen III mRNA in the heart were higher in MI rats than that in sham rats. The increase of collagen I and collagen III mRNA levels were reduced after miR-146a antagomiR treatment (Figure 4A).Treatment with miR-146a agomiR increased collagen I and collagen III mRNA levels in the heart of Sham rats. The increases of collagen I and collagen III mRNA levels were further enhanced after miR-146a agomiR treatment in MI rats (Figure 4B). Sirius Red staining showed that cardiac fibrosis level was increased in MI rat. The increase of cardiac fibrosis level was reduced after miR-146a antagomiR treatment. The increase of cardiac fibrosis level was further enhanced after miR-146a agomiR treatment in MI rats (Figure 4C).

### Effects of miR-146a in cardiomyocytes

**Figure 5:**
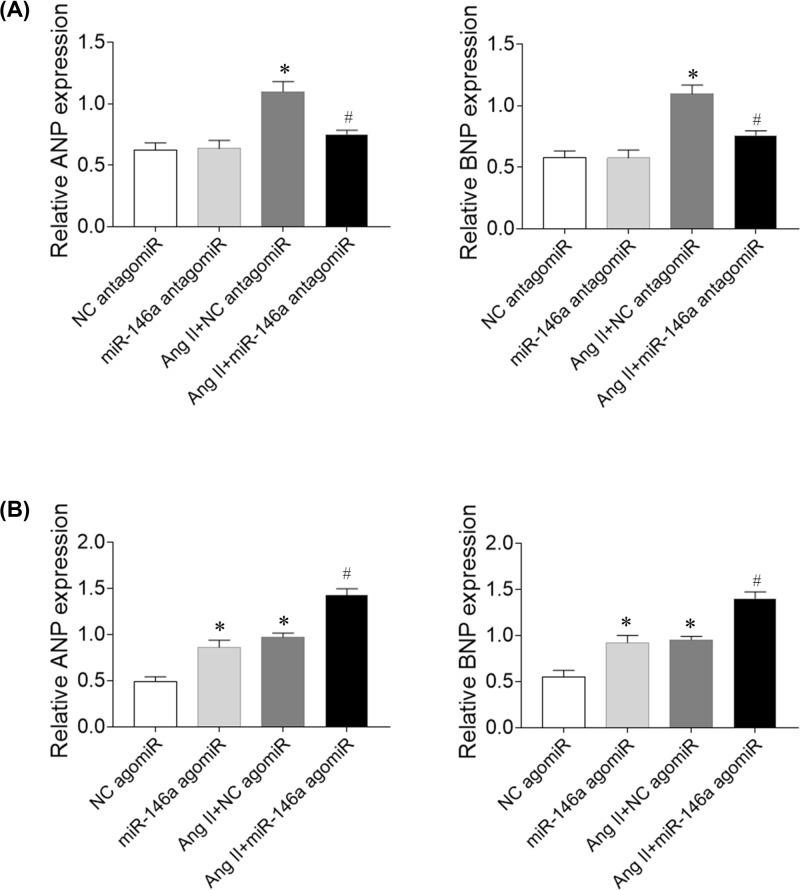
Effects of microRNA (miR)-146a on angiotensin (Ang) II-treated rat primary cardiomyocytes (**A**) miR-146a antagomiR reduced the increases of atrial natriuretic peptide (ANP) and brain natriuretic peptide (BNP) mRNA levels induced by Ang II in cardiomyocytes. (**B**) miR-146a agomiR further exacerbated the increases of ANP and BNP mRNA levels induced by Ang II in cardiomyocytes. The results are expressed as mean ± SEM. *P < 0.05 versus the negative control (NC) group; ^#^*P* < 0.05 versus the Ang II group.

Ang II increased ANP and BNP mRNA expression in cardiomyocytes. Treatment with miR-146a antagomiR inhibited the increases of ANP and BNP mRNA expression (Figure 5A). miR-146a agomiR exacerbated the increases of co ANP and BNP expression induced by Ang II administration in cardiomyocytes. Moreover, miR-146a agomiR increased ANP and BNP mRNA levels in normal cardiomyocytes (Figure 5B).

### Effects of miR-146a in CFs

**Figure 6:**
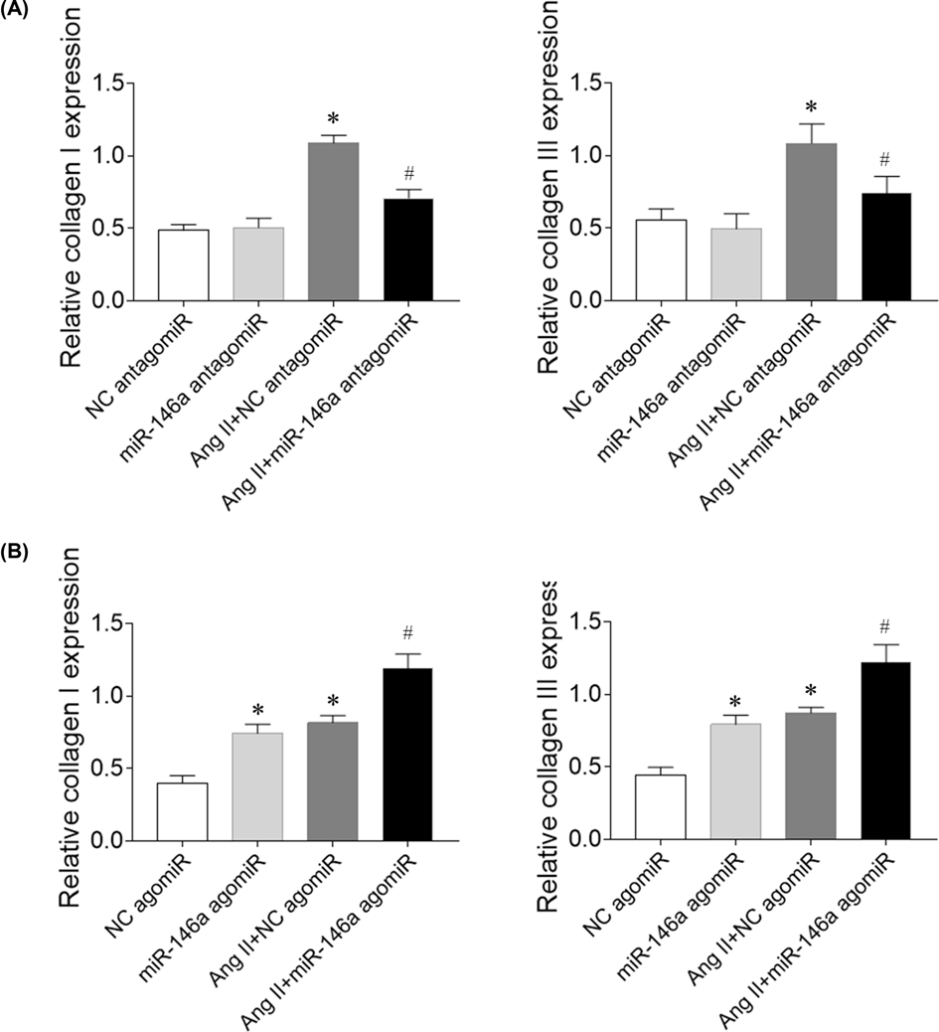
Effects of microRNA (miR)-146a on fibrosis induced by angiotensin (Ang) II in rat cardiac fibroblasts (CFs) (**A**) miR-146a antagomiR attenuated fibrosis induced by Ang II in rat CFs. (**B**) miR-146a agomiR exacerbated fibrosis induced by Ang II in rat CFs. The results are expressed as mean ± SEM. *P < 0.05 versus the negative control (NC) group; ^#^*P* < 0.05 versus the Ang II group.

Ang II increased collagen I and collagen III mRNA expression in CFs. Treatment with miR-146a antagomiR inhibited the increases of collagen I and collagen III mRNA expression (Figure 6A). miR-146a agomiR exacerbated the increases of collagen I and collagen III expression induced by Ang II administration in CFs. Moreover, miR-146a agomiR increased collagen I and collagen III mRNA levels in normal CFs (Figure 6B).

### Effects of miR-146a on Akt and ERK signaling pathways in HF rats

**Figure 7:**
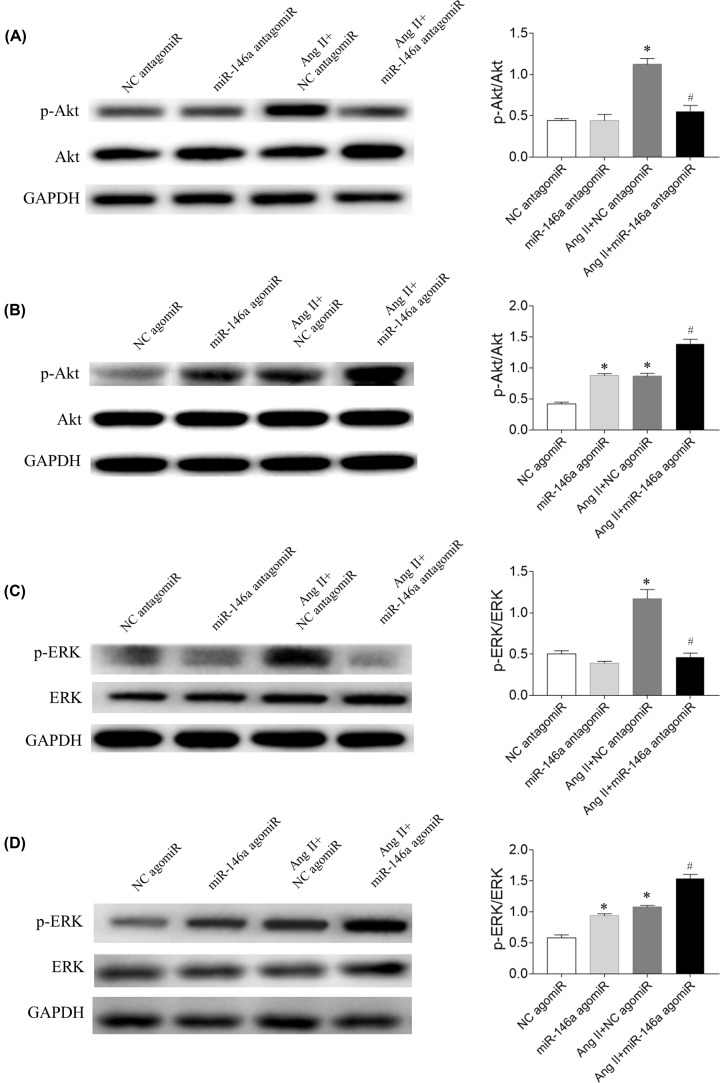
Effects of microRNA (miR)-146a on signaling pathway in rat cardiac fibroblasts (CFs) (**A**) miR-146a antagomiR inhibited p-Akt levels induced by angiotensin (Ang) II in rat CFs. (**B**) miR-146a agomiR exacerbated p-Akt levels induced by Ang II in rat CFs. (**C**) miR-146a antagomiR inhibited p-ERK levels induced by Ang II in rat CFs. (**D**) miR-146a agomiR exacerbated p-ERK levels induced by Ang II in rat CFs. The results are expressed as mean ± SEM. *P < 0.05 versus the negative control (NC) group; ^#^*P* < 0.05 versus the Ang II group.

The expression of p-Akt and p-ERK were increased in the heart of MI rats. Treatment with miR-146a antagomiR inhibited the increases in the p-Akt and p-ERK levels in the heart of MI rats, and miR-146a agomiR further worsen the increases in the p-Akt and p-ERK levels in the heart of MI rats (Figure 7).

## Discussion

MI-associated myocardial dysfunction leads to high mortality and morbidity in critical patients. Accumulating evidence has revealed the potential clinical values and the critical regulatory effects of multiple miRNAs in HF [22–24], although the precise mechanisms are far from clarified. miR-146a is a critical miRNA that regulates vascular smooth muscle cell apoptosis in a rat model of coronary heart disease [25], cardiac fibrosis [26], and is higher in human hearts of HF patients and in mouse hearts of transverse aortic constriction (TAC) operated mice [20]. However, the roles of miR-146a in MI-induced cardiac dysfunction and remodeling are still unclear. The present study demonstrated that inhibition miR-146a improved cardiac dysfunction and hemodynamics impairment, and attenuated cardiac remodeling in MI-induced HF rats.

Actually, the roles of miR-146a in cardiovascular disease are controversial depending on different experimental models. Adeno-associated virus serotype 9 mediated miR-146a overexpression reduced cardiac contractility, and miR-146a inhibition improved cardiac contractile function [20]. In contrast, transfection of LmiR-146a attenuated sepsis-induced cardiac dysfunction [19]. Therefore, it is highly needed to elucidate the functional roles of miR-146a in MI-cardiac dysfunction. In the present study, we found that MI induced reduction of FS (%) and EF (%), and increases of LVVd and LVVs. miR-146a antagomiR increased the reduction of FS (%) and EF (%), and reduced the increases of LVVd and LVVs in heart failure rats. Treatment with miR-146a agomiR reduced EF (%) and FS (%), and increased LVVd and LVVs in Sham rats. miR-146a agomiR aggravated the decreases of EF (%) and FS (%) in MI rats. Furthermore, the increases of LVVd and LVVs in MI rats were further enhanced by miR-146a agomiR injection. These results demonstrate that miR-146a inhibition improves cardiac dysfunction in MI-induced HF.

Cardiac hemodynamics were impaired in rats with CHF, including decrease of LVSP and +d*P*/d*t*_max_, and increase of LVEDP [21]. miR-146a might be a new and promising therapeutic tool for treating cardiac disorders [27]. Our data showed that LV +d*p*/d*t*_max_, LV −d*p*/d*t*_max_ and LVSP were decreased, and LVEDP was increased in MI rats, which were reversed by miR-146a antagomiR treatment. miR-146a agomiR reduced LV +d*p*/d*t*_max_, LV −d*p*/d*t*_max_ and LVSP in Sham rats. LVEDP was increased after miR-146a agomiR injected into Sham rat. Injection of miR-146a agomiR into the vein aggravated the decreases of LV +d*p*/d*t*_max_, LV −d*p*/d*t*_max_ and LVSP. The increase of LVEDP was reinforced after miR-146a agomiR treatment. These results indicated that the impaired cardiac hemodynamics was attenuated by miR-146a inhibition. LV +d*p*/d*t*_max_ and EF are both widely used and accepted parameters to assess cardiac contractility in HF. LVEF is a more sensitive marker of LV damage than d*P*/d*t*_max_ as previous study demonstrated [28]. In the present study, impaired cardiac function and cardiac hemodynamics were improved by miR-146a inhibition.

Circulating miR-146a may be a novel biomarker predictive of LV remodeling after acute MI [29]. Treatment with miR-146a antagomiR inhibited the increases of collagen I and collagen III mRNA expression in CFs, but miR-146a agomiR exacerbated the increases of collagen I and collagen III expression induced by Ang II in CFs. Treatment with miR-146a antagomiR inhibited the increases of ANP and BNP mRNA expression in cardiomyocytes, but miR-146a agomiR exacerbated the increases of ANP and BNP expression induced by Ang II in cardiomyocytes. Moreover, miR-146a agomiR increased collagen I and collagen III mRNA levels in normal CFs, and increased ANP and BNP mRNA expression in cardiomyocytes. The results demonstrated that miR-146a induced cardiac remodeling, and miR-146a inhibition attenuated cardiac remodeling in HF rats.

Chronic natriuretic peptides and sympathetic nerve system activation are major hallmark of adverse LV remodeling [30]. N-terminal pro-BNP (NT-pro-BNP) [31] and NE [32] are used as predictors for coronary disease in HF patients. Impressively, the present study revealed that the increases of ANP, BNP, ST2 and NE levels were reduced after miR-146a antagomiR treatment in MI rats. Treatment with miR-146a agomiR increased ANP, BNP, ST2 and NE levels in Sham rats. The increases of ANP, BNP, ST2 and NE levels were further enhanced after miR-146a agomiR treatment in MI rats. These results demonstrated that miR-146a inhibition attenuated neurohormonal activation in HF.

The key role of the Akt signaling pathway has been well generally acknowledged in cardiac hypertrophy [33,34]. Inhibition of Akt signaling reverses the hypertrophic phenotype *in vivo* and *in vitro* [35]. Studies showed that the ERK signaling pathway is involved in cardiac fibrosis and hypertrophy [36,37]. In the present study, we found that the levels of p-Akt and p-ERK were increased in the heart of MI rats, which were improved by treatment with miR-146a antagomiR and were further worsened by miR-146a agomiR. These results indicated that miR-146a antagomiR improved cardiac remodeling by inhibiting Akt and ERK signaling pathway.

The limitation of the present study is that the mechanism of inhibition of miR-146a on improving cardiac dysfunction and inhibiting cardiac remodeling in heart failure is not explored in the present study. Study shows that miR-146a reduced small ubiquitin-like modifier 1 (SUMO1) expression, sarcoplasmic reticulum Ca^2+^-ATPase (SERCA2a) SUMOylations, and thus worsened calcium cycle in cardiomyocytes and worsened contractility [20]. Fos is a direct target of miR-146a activity and that down-regulation of the Fos-AP-1 pathway by miR-146a has the capacity to inhibit matrix metalloproteinase (MMP)-9 activity [27]. miR-146a regulates cholesterol metabolism and tempers chronic inflammatory responses to atherogenic diet by restraining proinflammatory signaling in endothelial cells and BM-derived cells [38]. Moreover, mir-146a levels can affect the NETosis process in relation to the occurrence of adverse cardiovascular events in patients with atrial fibrillation [39]. The mechanism of miR-146a regulating cardiac remodeling in heart failure is an interesting issue and will be explored in our future study.

In conclusion, the present study shows that inhibition of miR-146a improves cardiac function and hemodynamics, and reduces remodeling in hearts with HF. miR-146a antagomiR attenuated fibrosis of CFs. The miR-146a pathway might be a potential novel therapeutic target for myocardial dysfunction in HF.

## Abbreviations

Ang: angiotension
ANP: atrial natriuretic peptide
BNP: brain natriuretic peptide
CF: cardiac fibroblast
CLP: cecal ligation and puncture
CVD: cardiovascular disease
EF: ejection fraction
FS: fractional shortening
HF: heart failure
LV: left ventricle
LVEDP: left ventricle end-diastolic pressure
LVVd: left ventricle volumes in diastole
LVSP: left ventricle systolic pressure
LVV: left ventricle volume
MI: myocardial infarction
miR: microRNA
SD: Sprague-Dawley
